# Intention to use and acceptability of home-based sexual health care among men who have sex with men who previously attended clinic-based sexual health care

**DOI:** 10.3389/frph.2022.967770

**Published:** 2022-08-15

**Authors:** Cornelia J. D. Goense, Ymke J. Evers, Christian J. P. A. Hoebe, Rik Crutzen, Nicole H. T. M. Dukers-Muijrers

**Affiliations:** ^1^Department of Sexual Health, Infectious Diseases and Environmental Health, South Limburg Public Health Service, Heerlen, Netherlands; ^2^Department of Social Medicine, Care and Public Health Research Institute (CAPHRI), Maastricht University, Maastricht, Netherlands; ^3^Department of Medical Microbiology, Care and Public Health Research Institute (CAPHRI), Maastricht University Medical Centre (MUMC+), Maastricht, Netherlands; ^4^Department of Health Promotion, Care and Public Health Research Institute (CAPHRI), Maastricht University Medical Centre (MUMC+), Maastricht, Netherlands

**Keywords:** HIV testing, men who have sex with men (MSM), sexually transmitted infections (STI), self-sampling, home-based sexual health, COVID-19

## Abstract

The COVID-19 pandemic has temporarily disrupted access to clinic-based sexual health care for men who have sex with men (MSM) in the Netherlands. The importance of home-based sexual health care has been underpinned as an extension of clinic-based care. This paper aims to assess intention to use, and acceptability of home-based sexual health care among MSM who previously attended clinic-based sexual health care. In November 2020, 424 MSM who had attended an STI clinic pre-pandemic were invited to participate in an online survey; 154 MSM completed the survey (response 36%). Intention to use self-sampling STI/HIV tests was assessed (median; scale 0–100) and compared across sociodemographic and sexual behavior characteristics by Kruskal-Wallis H tests. Descriptive analyses provided insights in acceptability of home-based sexual health care. Of participants (median age 47), 60.4% (93/154) tested for STI/HIV in the past 6 months, most of them attended a clinic. The median score on intention to use self-sampling tests was 86.5 (SD = 33.4) and did not differ by sociodemographic or sexual behavioral characteristics (all *p*-values > 0.1). Participants were positive toward online sexual health counseling (median attitude = 75.0, SD = 29.6) and their main preferred topics were PrEP use and STI/HIV testing. MSM who attended clinic-based care expressed intention to use self-sampling tests and a positive attitude toward online sexual health counseling. Home-based sexual health care elements are not currently integrated within Dutch clinic-based sexual health care and should be considered an addition for continued provision of care and extended reach of MSM.

## Introduction

Due to the COVID-19 pandemic, men who have sex with men (MSM) in the United States and China reported difficulties in access to testing services for sexually transmitted infections (STI) and human immunodeficiency virus (HIV) (1, 2). Disruptions in access to sexual health care services were reported in many other countries, including in the Netherlands (3). Lower testing rates were observed in a context where MSM remained sexually active during lockdown situations and social distancing measures in the Netherlands, as to the United Kingdom reported sexual activity remained high (4).

In recent years, new opportunities for STI/HIV testing include commercial home-based alternatives, which include self-tests and self-sampling tests (5). To extend clinic-based care, home-based sexual health care could be a cost-effective possibility when compared with health care in a clinic, as demonstrated in the United Kingdom (6–8). Home-based sexual health care consists of self-collection of samples, and additional online sexual health counseling, for example about PrEP and chemsex. Home-based alternatives for testing can overcome perceived and experienced barriers among MSM for STI/HIV testing at STI clinics such as privacy, confidentiality, and time-constraints (9, 10). Home-based care options enable continual access to care for MSM who seek sexual health care, and due to COVID-19 restrictions, personal needs, or other reasons, experience barriers to clinic-based testing or sexual health counseling. Insights into added value remains limited on combining STI and HIV tests in complete home-based sexual health care, with high quality STI/HIV diagnostics and additional sexual health care opportunities, such as sexual health counseling on safe sex, PrEP and, chemsex (11, 12). An ongoing study from the United States identified a lack of counseling additional to home-based testing among male couples (13), but acceptability of sexual health counseling was not specified.

Self-sampling STI and HIV tests are provided by commercial initiatives in the Netherlands, with the main challenge being the lack of provision of complete sexual health care services, i.e., including testing for both STI and HIV, sexual health counseling, treatment, and partner services (14). Such comprehensive sexual health care is provided by location-based STI clinics. Self-collection of samples for STI and HIV testing outside of a clinical setting has not yet been implemented in sexual health care by Dutch STI clinics (15, 16).

The current study assessed the opportunities for home-based sexual health care in MSM who previously attended clinic-based sexual health care. Therefore, we assessed the intention, behavioral determinants (e.g., attitude, social norms, self-efficacy) and acceptability of self-sampling testing and online sexual health counseling.

## Materials and methods

### Data collection

In the Netherlands, STI/HIV testing and sexual health care is organized *via* STI clinics (Public Health Services), offering free of charge care to high-risk groups, including MSM. Other care options include the general practitioner or hospital, though here patients may need to pay for STI and HIV tests. In November 2020 we invited 424 MSM by e-mail to participate in an online survey. After two reminders were sent, respondents were able to complete the survey until February 2021. This was a cross-sectional study with a convenience sample. Respondents in this study had participated in a previous study before the COVID-19 pandemic (2018) and were recruited during their regular consultation by an STI nurse in one of nine STI clinics in the Netherlands (Supplementary Table S1). The invited study cohort consisted of respondents who previously consented to participate in future studies (Supplementary Table S2). Respondents were eligible to participate if they reported to be male and had sex with other men.

### Measurements

The survey assessed STI/HIV testing behavior, intention to use self-sampling testing, related behavioral determinants, and acceptability of home-based sexual health care. Furthermore, sexual behavior, PrEP and drug use behavior were assessed. Sociodemographic information such as age, level of education and ethnicity were available from pseudo-anonymized data from the STI clinic consultation. Upon consent at the start of the survey, STI clinic data was linked to the survey data by a code.

#### STI/HIV testing behavior

Participants reported when and where they had tested for STI and HIV in the past 6 months. STI/HIV testing is defined as testing urogenital, anorectal, and oropharyngeal Chlamydia trachomatis (CT), Neisseria gonorrhoeae (NG), syphilis or HIV. A multiple response question assessed where participants tested the last time (e.g., at general practitioner, STI clinic, hospital, or, by a self-sampling test). Self-sampling testing was defined as self-collection of specimens analyzed by a laboratory for the results, whereas results of self-testing is interpreted by the individual (5). The survey defined self-sampling STI and HIV testing as “taking a urine sample, anorectal and oropharyngeal swabs and blood sampling by fingerstick”. Online sexual health counseling was defined as counseling by a health care professional *via* videocalls, chat, or a telephone call. Participants were presented with a context–in line with current Dutch practice–where these services would be available without costs and offered by the Public Health Service.

#### Intention to use a self-sampling test

Use of a self-sampling STI/HIV test is seen as behavior, which is determined by intention, behavioral beliefs, attitude, efficacy beliefs and normative beliefs of the individual (17, 18). In line with explanatory theory regarding behavior (e.g., Theory of Planned Behavior, Reasoned Action Approach) Table 1 shows items and measures to capture determinants of self-sampling testing.

**Table 1:** Measurement of behavioral determinants of self-sampling STI/HIV testing.

**Determinant**	**Question**	**Answering scale**	**Answering options**
Intention	“*Would you use a self-sampling test for STI/HIV?”*	Interval (Scores 0–100)	(0) Definitely not (100) Definitely^$^
Attitude	“*I am negative/positive toward self-sampling testing”*	Interval (Scores 0–100)	(0) Negative (100) Positive
Self-efficacy	“*Do you think you are capable of performing a self-sampling test?” “Self-sampling tests seem difficult/easy”*	Interval (Scores 0–100)	(0) Definitely not (100) Definitely (0) Difficult (100) Easy
Behavioral beliefs	“*The thought of self-sampling testing makes me anxious/reassured”*	Interval (Scores 0–100)	(0) Anxious (100) Reassured
Social support	“*Most of the people whose opinion I value, would not support me/ support me a lot when I use a self-sampling test”*,	Interval (Scores 0–100)	(0) Not support me (100) Support me a lot
Subjective norm	“*Most of the people whose opinion I value, would disapprove/approve of self-sampling testing”*	Interval (Scores 0–100)	(0) Disapprove (100) Approve
Descriptive norm	“*Most of my male sex partners or male friends would use self-sampling tests”*	Interval (Scores 0–100)	(0) Nobody (100) Everybody

$ High intention is defined when scored over 80, low intention scored up to 80.

#### Acceptability

Acceptability of self-sampling STI/HIV testing were assessed in mode of access (e.g., home delivery or pick-up location), receiving instructions how to use the self-sampling tests (e.g., *via* website, *via* peers, through telephone counseling). Whether participant were positive toward online sexual health counseling was measured from “negative” (0) to “positive” (100) by the question “are you negative/ positive toward online sexual health counseling”. Acceptability of modes of communication for online sexual health counseling (e.g., telephone call, webcam, or chat) and what topics to discuss (e.g., chemsex, PrEP use) were assessed using multi response questions (Supplementary Tables S3, S4).

#### Sociodemographic and sexual behavioral characteristics

For current analyses, age groups and numbers of sex partners were defined based on tertile distributions. Categories for ethnicity and level of education were based on definitions of the Central Bureau of Statistics in the Netherlands (www.//cbs.nl). Sexual behavioral data included number of sex partners, condomless anal intercourse (CAI) with a casual partner, HIV status, use of PrEP, engagement in chemsex, and STI/HIV testing in the past 6 months. For analyses, CAI was defined as either receptive or insertive anal intercourse without a condom. Casual partners included fuckbuddies, partners of whom they did not know their names, participants of group sex, customers (of sex work), sex workers, and friends. Chemsex was defined as having used one or more of the following drugs: *crystal meth, cocaine, 2-CB, 3MMC, 4-FA, 4-MEC, GHB, GBL, ketamine, MDMA, mephedrone, speed and XTC*, before or during sex in the past 6 months (19).

### Statistical methods

Characteristics of the study population were presented by descriptive analyses; a Chi-square test compared these characteristics among respondents' and non-respondents' characteristics of the invited study cohort. Subsequently, a Chi-square test was used to compare sociodemographic and sexual behavioral characteristics between recent (past 6 months) and non-recent testers (more than 6 months ago). Self-sampling and participants' behavioral determinants, i.e., intention to use self-sampling tests were presented using descriptive statistics. Attitude, self-efficacy, and intention showed strong (>0.7) Pearson correlations, hence the strongest predictor for behavior according to Theory of Planned Behavior/Reasoned Action Approach (i.e., intention) was chosen as outcome to assess differences between population subgroups. A Shapiro-Wilk test revealed that intention to use self-sampling testing was not normally distributed (*p* = 0.000). Therefore, a non-parametric Kruskal-Wallis H test was used to compare median intention across sociodemographic and sexual behavioral subgroups. Last, acceptability was demonstrated by descriptive analyses and a Chi-Square test compared several (dis)advantages among participants with low and high intention to use a self-sampling test. All analyses were performed using IBM SPSS Statistics V26.

## Results

Of 424 invited MSM who previously attended a Dutch STI clinic, 154 MSM participated (response rate of 36%). Participants in this study were mostly of western ethnicity (95.5%; 147/154) and 63% (97/154) were highly educated, median age was 47 (IQR = 22.2) (Table 1). Majority of participants were HIV negative (81.8%; 126/154), 37.7% (58/154) reported PrEP use and 37.0% (57/154) engagement in chemsex in the past 6 months. Median number of sex partners was five (IQR = 8) and CAI with a casual partner was reported by 59.7% (92/154).

### STI/HIV testing behavior

During the COVID-19 pandemic, 60.4% (93/154) of the participants tested for STI or HIV (mostly for both) in the past 6 months, 23.4 % (36/154) indicated to have tested more than 12 months ago. Of participants who tested in the past 6 months (recent), 84.9% (79/93) tested at the STI clinic (56.5%; 87/154), 9.7% (9/93) have tested at the GP or at a hospital (9.7%; 9/93). Only 3.2% (3/93) reported self-sampling STI or HIV testing, whereas no participant used a self-test. Among recent STI/HIV testing MSM a higher number of sex partners, CAI with a casual partner, PrEP use or chemsex were reported (Table 2). Among non-recent testing MSM, 60.6 % (37/61) reported more than four sex partners, 24.6 % (14/61) chemsex, and 36.1% (22/61) CAI with a casual partner.

**Table 2:** Characteristics study sample of men who have sex with men who previously attended clinic-based sexual health care in the Netherlands.

		**Recent STI/HIV test (past 6 months)**		
	**Total (*N =* 154)**	**Yes (*N =* 93)**	**No (*N =* 61)**	***p***
	**% Of total (n)**	**% Within group (n)**	**% Within group (n)**	
**Ethnicity^a^**				0.332
Western	95.5 (147)	96.8 (90)	93.4 (57)	
Non-western	4.5 (7)	3.2 (3)	6.6 (4)	
Education^a^^*^				0.393
High	63.0 (97)	70.6 (60)	63.8 (37)	
Low	29.9 (46)	29.4 (25)	36.2 (21)	
**Age^b^**				0.361
15–42 years	31.8 (49)	28.0 (26)	37.7 (23)	
43–54 years	37.0 (57)	40.9 (38)	31.1 (19)	
55 + years	31.2 (48)	31.2 (29)	31.1 (19)	
**No. sex partners^b^^c^**				**<0.0001**
0–3	37.7 (58)	14.0 (13)	39.3 (24)	
4–8	27.9 (43)	30.1 (28)	34.4 (21)	
8 +	34.4 (53)	**55.9 (52)**	26.2 (16)	
**CAI with casual partner^c^**				**<0.0001**
Yes	59.7 (92)	**75.3 (70)**	36.1 (22)	
No	40.3 (62)	24.7 (23)	63.9 (39)	
HIV status^*^				**0.004**
Positive	16.2 (25)	9.7 (9)	27.6 (16)	
Negative	81.8 (126)	**90.3 (84)**	72.4 (42)	
**PrEP use**				**<0.0001**
Yes	37.7 (58)	**58.1 (54)**	6.6 (4)	
No	62.3 (96)	41.9 (39)	93.4 (57)	
**Chemsex^c^**				**0.003**
Yes	37.0 (57)	**46.2 (43)**	23.0 (14)	
No	63.0 (97)	53.8 (50)	77.0 (47)	

a Ethnicity and level of education were based on definitions used by Central Bureau of Statistics (NL) (www.//cbs.nl). Middle level of education is classified as highly educated.

b Age groups and number of sex partners were based on tertile distributions.

c In the past 6 months.

* Education and HIV status do not count to 100 % due to missing data in education of 7.1% and 1.9 % that did not want to declare their HIV status. Bold values are statistically significant values.

#### Intention to use a self-sampling test

**Figure 1:**
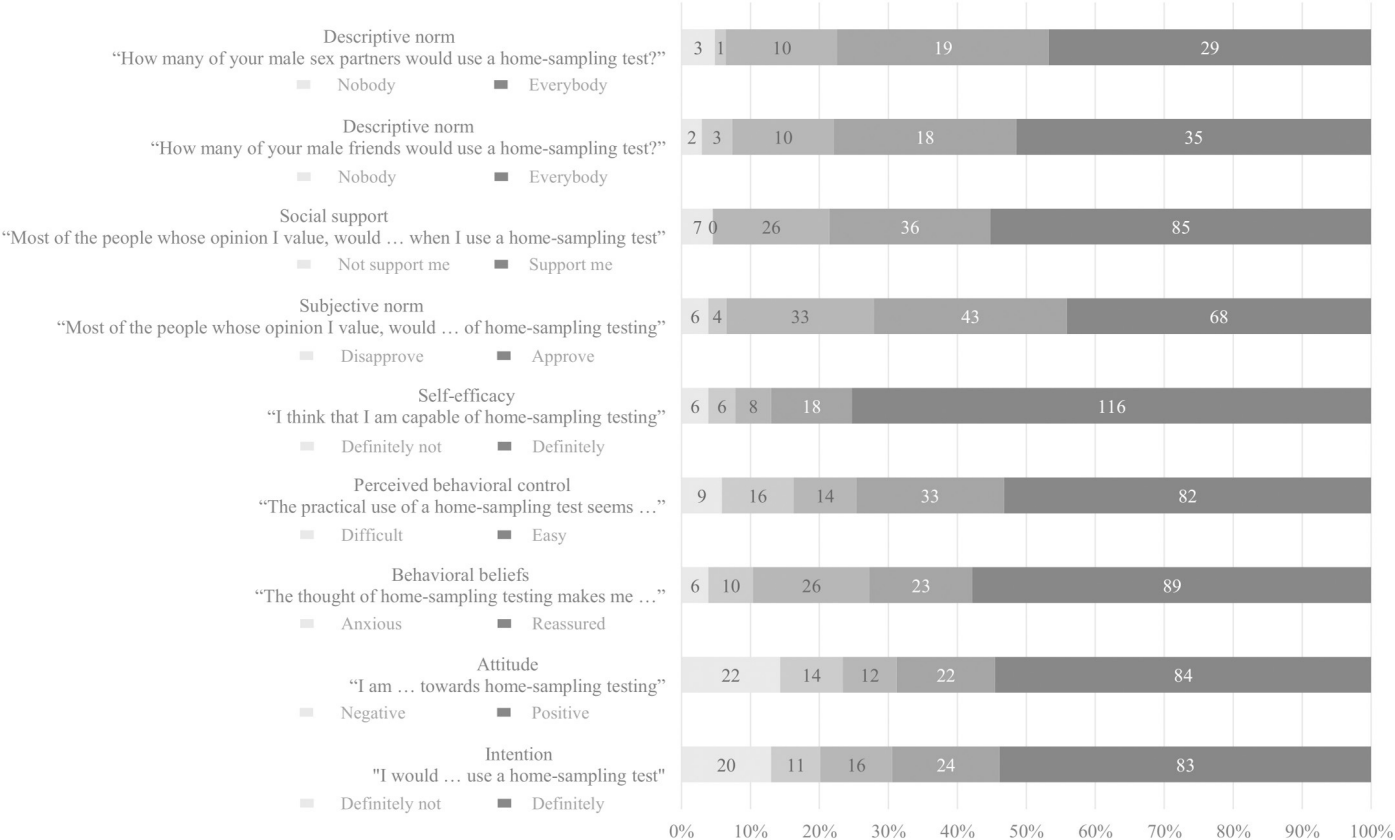
Behavioral determinants of self-sampling STI/HIV testing among MSM who previously attended clinic-based sexual health care in the Netherlands.

Figure 1 presents scores from 1–100 on behavioral determinants. Score of intention to use self-sampling tests was 86.5 (IQR = 50) and participants had positive attitude toward self-sampling testing (median = 88.5, IQR = 50). In addition, participants felt capable of performing a self-sampling test (median = 100, IQR = 19), and they perceived self-sampling to be easy (median = 83.5, IQR = 42). The thought of performing self-sampling test made them reassured (median = 90, IQR = 42). Scores of expected social support of people whose opinion they value was 87 (IQR = 34) and 79 (IQR=44) for the expected approval of these people. Participants estimated that most of their male friends (median = 81, IQR = 29) and male sex partners (median = 77.5, IQR = 32) would use a self-sampling test.

The median scores on intention to use self-sampling testing were not statistically significantly different by ethnicity, age, level of education or, by sexual behavioral characteristics (all *p*-values > 0.1) (Supplementary Table S5).

### Acceptability of home-based sexual health care

#### Self-sampling STI/HIV testing

Table 3 shows perceived advantages and disadvantages of self-sampling testing. Most reported advantages were saving time (77.9%; 120/154) and autonomy in deciding when to test (76.6%; 118/154). Participants with a high intention (53.9%; 83/154) to use a self-sampling test, reported these advantages more in comparison to participants with low intention. Perceived disadvantages included having to take the blood sample by themselves (57.8%; 103/154), and lack of a supporting conversation with a health care professional at a clinic (48.7%; 75/154). Participants with a low intention (46.1%; 71/154) to use a self-sampling test more often reported concerns about performing the test correctly.

**Table 3:** Perceived advantages and disadvantages of self-sampling testing compared between low or high intention to use self-sampling testing.

	**Total (*N =* 154)**	**Low intention** **(*N =* 71)**	**High intention (*N =* 83)**	***p***
	**% Of total (n)**	**% Within group (n)**	**% Within group (n)**	
**Perceived advantages**				
Saves time^$^	77.9 (120)	64.8 (46)	**89.2 (74)**	**<0.0001**
Saves money^$^	25.3 (39)	21.1 (15)	28.9 (24)	0.268
Determine when to test	76.6 (118)	59.2 (42)	**91.6 (76)**	**<0.0001**
Avoid encounter acquaintances^*^	14.9 (23)	9.9 (7)	19.3 (16)	0.102
Avoid encounter others^*^	9.1 (14)	7.0 (5)	10.8 (9)	0.413
No physical examination^&^^*^	2.6 (4)	0 (0)	4.8 (4)	0.061
No supporting conversation^&^^*^	4.5 (7)	1.4 (1)	7.2 (6)	0.084
Other	11.7 (18)	16.9 (12)	7.2 (6)	0.063
**Perceived disadvantages**				
Take the blood sample myself	57.8 (89)	57.7 (41)	57.8 (48)	0.992
Responsible correct testing	44.8 (69)	**62.0 (44)**	30.1 (25)	**<0.0001**
Responsible order and return of test	14.9 (23)	22.5 (16)	8.4 (7)	0.014
No physical examination^&^^*^	31.8 (49)	36.6 (26)	27.7 (23)	0.237
No supporting conversation^&^^*^	48.7 (75)	54.9 (39)	43.4 (36)	0.153
Other	9.7 (15)	9.9 (7)	9.6 (8)	0.963

$ For traveling to the STI clinic, or clinic-based facility.

* At the STI clinic, or clinic-based facility.

& Performed by a health care professional. Bold values are statistically significant values.

As to obtaining self-sampling tests, 79.2% (122/154) of participants preferred home delivery, 38.3% (59/154) *via* pick-up location (e.g., at a STI clinic, GP, or hospital), and 3.2% (5/154) to receive the test from a peer-friend or sex partner. Accompanying self-sampling tests, 33.1% (51/154) was unsure whether additional instructions were necessary, 33.8% (52/154) reported to probably need additional instructions and mentioned further instructions *via* a website (74.8%; 77/103), *via* support of a health care professional (52.4%; 54/103), or 5.8% (6/103) from peers. Of all participants, 83.1% (128/154) would recommend self-sampling STI/HIV testing to other MSM. Two thirds of participants would recommend it to a fuckbuddy (68%; 87/128), followed by a casual sex partner of whom they know their name (60.9%; 78/128). Only 22.7% (29/128) would recommend self-sampling tests to chat friends.

#### Online sexual health counseling

**Figure 2:**
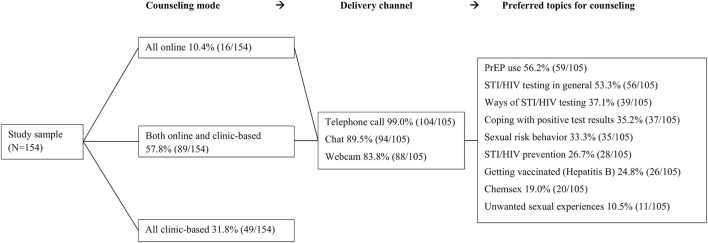
Acceptability of online sexual health counseling among MSM who previously attended clinic-based sexual health care in the Netherlands.

Figure 2 shows acceptability of online sexual health care. Participants demonstrated a positive attitude toward online sexual health counseling (median = 75, IQR = 48). Of all participants 10.4% (16/154) preferred all sexual health counseling to be online, over half of participants (57.8%; 89/154) preferred online counseling in combination with alternate clinic-based counseling. They preferred various modes of communication for counseling, including telephone contact (99.0%; 104/105), 89.5% (94/105) online chat or 83.8% (88/105) webcam counseling. Preferred topics to discuss during the online counseling included PrEP use (56.2%; 59/105), STI/HIV testing in general (53.3%; 56/105), and how to perform STI/HIV testing (37.1%; 39/105).

## Discussion

This study provided insight into intention to use and acceptability of home-based sexual health care for MSM who previously attended clinic-based sexual health care. Most participants tested for STI or HIV clinic-based during the COVID-19 pandemic. Recent testing was higher in MSM who had multiple sex partners, had CAI with a casual partner, used PrEP, and engaged in chemsex in the past 6 months. Most participants recently tested in clinics, and they expressed a high intention to future use of (non-clinic based) self-sampling STI/HIV tests. These results did not differ between population subgroups. Self-sampling tests are preferably ordered online with home delivery, their instructions are equally preferred online. In addition, a positive attitude toward online sexual health counseling was found and topics most often preferred to be addressed in online counseling were PrEP use and ways of STI/HIV testing. We showed acceptability of home-based care to continue reaching MSM who attended clinic-based care. Preferably, home-based care would also be able to extend the reach of sexual health care, by decreasing testing barriers. More research is needed to assess whether home-based sexual healthcare increases the reach of sexual health care in populations who have no previous experience with attending sexual health care.

The uptake of STI/HIV tests has decreased among MSM during the COVID-19 pandemic, as a result of downscaling clinic-based sexual health care and stricter triaging (3). Therefore, this pandemic underlined the need for home-based sexual health care, including STI/HIV testing for MSM (1, 2). Global studies have reported high feasibility and acceptability among MSM of testing outside a clinic (20–22). The current study demonstrated intention to use self-sampling STI/HIV testing in a population of MSM who previously attended clinic-based sexual health care. Additionally, we established a positive attitude toward online sexual health counseling. Several studies have suggested integration of home-based care in existing sexual health care to increase STI/HIV testing uptake and ensure quality of sexual health care (10, 11, 23). Particularly a recent study from the United States, which modeled an increase of HIV incidence when testing in clinical setting is completely replaced by home-based testing (24). Thus, home-based sexual health care should be considered an extension of existing clinic-based care.

Previous studies have emphasized minor variation in sociodemographic characteristics (e.g., age, education) among intention to use STI/HIV testing outside a clinical setting (25, 26). Consistent with results in this paper, we previously established a positive attitude toward self-sampling testing in a cohort of Dutch HIV-positive MSM (16). Therefore, home-based sexual health care should be designed for a diverse and extensive group of MSM who previously attended clinic-based care. An inclusive approach should be accomplished by tailored home-based sexual health care, which would provide with free and easy-to-use tests to tackle perceived barriers (26).

Implications from this study should be interpreted considering respondents' previous experience with clinic-based sexual health care. Home-based care should not solely be designed to extend the reach of existing sexual health care, also to continue reaching MSM who attended clinic-based care before (27). Since participants mainly identified internet-based options, home-based sexual health care for MSM should provide with online opportunities. Fitting home-based care would consist of ordering self-sampling STI/HIV tests online accompanied with online instructional content. Consequently, previous research recommends use of illustration and detailed verbal instructions when self-sampling testing (28). Within home-based care, online sexual health counseling should be available as an additional option to clinic-based counseling and could take place by telephone call, *via* a chat function, or webcam managed by a health care professional. In addition, previous studies have suggested the use of mobile apps as a mode of communication for home-based sexual health care (22, 29). Within online sexual health care counseling topics such as PrEP use and STI/HIV testing could be discussed. Furthermore, home-based sexual health care could be considered a cost-efficient option, (6, 8) therefore financial allocations can prioritize reaching out to higher-risk groups who are hard to reach with existing sexual health care. Future research should also include assessing possibilities for home-based sexual health care for other key populations such as transgender people and sex workers.

### Limitations

This study has several limitations. First, response for this follow-up study sample (36.3%; *N* = 154) was lower than anticipated. Yet, the sample may be only minimally subject to bias due to loss-to-follow-up as proportions of sociodemographic and sexual behavioral characteristics were similar to the invited study cohort (Supplementary Table S2). Second, the invited study sample included MSM with somewhat higher sexual risk behavior, the median number of sex partners (6 vs. 5, *p* = 0.02) and STI positivity rate were higher (23 vs. 19%, *p* = 0.02) compared to total MSM population who visited the participating STI clinics (19). This slightly affects the generalizability of the results of the study to all MSM who visit an STI clinic. However, we expect that impact on generalizability is minimal as intention to use self-sampling testing was not associated with numbers of sex partners and STI outcome. Generalizability of the results to MSM who did not visit an STI clinic is unknown, but it is possible that the intention to use home-based care and positive attitude toward home-based sexual health care might be lower in this group. Third, at the time of the study integration of home-based sexual health care elements were limited within Dutch STI clinics. Therefore, this study assessed behavioral determinants rather than actual use of self-sampling STI/HIV testing (30). Fourth, the quantitative nature of this study did not allow elaborate qualitative reporting on acceptability. Nevertheless, this study did provide detailed information on acceptability of home-based sexual health care among MSM who previously attended clinic-based sexual health care.

## Conclusion

Although current self-sampling STI/HIV testing is rarely used by MSM who previously attended clinic-based sexual health care, results present that MSM would consider future use of a self-sampling STI/HIV test. Home-based sexual health care including self-sampling STI/HIV testing and online sexual health counseling might be a possibility to extend sexual health care to continue servicing MSM with proper sexual health care.

## Data availability statement

The raw data supporting the conclusions of this article will be made available by the authors, without undue reservation.

## Ethics statement

The studies involving human participants were reviewed and approved by Medical Ethics Committee of Maastricht University Medical Centre (MUMC+). The patients/participants provided their written informed consent to participate in this study.

## Author contributions

CG, YE, RC, and ND-M contributed to conception and design of the study. CG, YE, and ND-M performed the statistical analysis. CG wrote the first draft of the manuscript. All authors contributed to manuscript revision, read, and approved the submitted version.

## Funding

This study received funding from Aidsfonds Nederland under Grant P-49903. The funder was not involved in the study design, collection, analysis, interpretation of data, the writing of this article, or the decision to submit it for publication.

## Conflict of interest

The authors declare that the research was conducted in the absence of any commercial or financial relationships that could be construed as a potential conflict of interest.

## Publisher's note

All claims expressed in this article are solely those of the authors and do not necessarily represent those of their affiliated organizations, or those of the publisher, the editors and the reviewers. Any product that may be evaluated in this article, or claim that may be made by its manufacturer, is not guaranteed or endorsed by the publisher.
