# Supplementary material for: Intention to use and acceptability of home-based sexual health care among men who have sex with men who previously attended clinic-based sexual health care
Source: Front Reprod Health. 2022 Aug 15;4:967770. doi: 10.3389/frph.2022.967770 (PMC9580753; doi:10.3389/frph.2022.967770)
Supplement: Supplementary file 1 [file Table_1.pdf]

Supplementary Table S1. List of participating STI clinics

GGD Zuid-Limburg

GGD Limburg Noord

GGD Brabant Zuid-Oost

GGD Hart voor Brabant

GGD Gelderland-Midden

GGD Gelderland-Zuid

GGD Twente

GGD Haaglanden

GGD Ijsselland
