# Supplementary material for: Intention to use and acceptability of home-based sexual health care among men who have sex with men who previously attended clinic-based sexual health care
Source: Front Reprod Health. 2022 Aug 15;4:967770. doi: 10.3389/frph.2022.967770 (PMC9580753; doi:10.3389/frph.2022.967770)
Supplement: Supplementary file 2 [file Table_2.pdf]

Supplementary Table S2. Characteristics invited study cohort and study sample of MSM who previously attended clinic-based sexual health care in the Netherlands

	Invited cohort (N=424)	Study sample (N=154)	*p*
	**% Of total (n)**	**% Of total (n)**	
**Ethnicity** [a] [*]			.246
Western	90.8 (385)	95.5 (147)	
Non-western	6.6 (28)	4.5 (7)	
**Education** [a] [*]			.064
High	64.2 (272)	59.1 (97)	
Low	27.8 (118)	29.9 (46)	
**Age** [b] [*]			.047
15 – 42 years	58.5 (248)	31.8 (49)	
43 – 54 years	21.7 (92)	37.0 (57)	
55 + years	17.2 (73)	31.2 (48)	
**No. sex partners** [b c] [*]			**.009**
0 – 3	24.5 (104)	37.7 (58)	
4 – 8	33.3 (141)	27.9 (43)	
8 +	36.6 (155)	34.4 (53)	
**HIV status** *			**.003**
Positive	14.4 (61)	16.2 (25)	
Negative	84.9 (360)	81.8 (126)	
**PrEP use**			.053
Yes	12.7 (54)	37.7 (58)	
No	87.3 (370)	62.3 (96)	
**Chemsex** [c]			**.003**
Yes	42.0 (178)	37.0 (57)	
No	58.0 (246)	63.0 (97)	
**STI/HIV testing**			-
< 6 months		60.4 (93)	
6 – 12 months		16.2 (25)	
12 months +		23.4 (36)	

[a] Ethnicity and level of education were based on definitions used by Central Bureau of Statistics (NL) (www.//cbs.nl). Middle level of education is classified as highly educated.

[b] Age groups and number of sex partners were based on tertile distributions

[c] In the past six months

[*] Ethnicity, education, age, no. of sex partners and HIV status do not count to 100 %, due to missing values in the invited cohort for ethnicity 2.6%, education 8%, age 2.6%, no. of sex partners 5.7% and 0.7% did not declare HIV status. For the study sample education and HIV status do not count to 100 % due to missing educational level of 7.1% and 1.9 % that did not want to declare their HIV status
