# Supplementary material for: Intention to use and acceptability of home-based sexual health care among men who have sex with men who previously attended clinic-based sexual health care
Source: Front Reprod Health. 2022 Aug 15;4:967770. doi: 10.3389/frph.2022.967770 (PMC9580753; doi:10.3389/frph.2022.967770)
Supplement: Supplementary file 3 [file Table_3.pdf]

Supplementary Table S3. Questions to determine behavioral determinants of self-sampling STI/HIV testing

- "I would use a home-sampling test"
  0 Definitely not – 100 Definitely

- "I am … towards home-sampling tests"
  - 0 Negative – 100 Positive

- "The practical use of a home-sampling test seems …"
  - 0 Difficult – 100 Easy

- "I think I am capable of performing a home-sampling test"
  - 0 Definitely not – 100 Definitely

- "The thought of home-sampling testing makes me …"
  - 0 Anxious – 100 Reassured

- "Most of the people whose opinion I value, would … of home-sampling testing"
  - 0 Disapprove – 100 Approve

- "Most of the people whose opinion I value, would … when I use a home-sampling test"
  - 0 Not support me – 100 Support me a lot

- How many of your male friends (who have sex with men) would use a home-sampling test?
  - 0 Nobody – 100 Everybody
- How many of your male friends (who have sex with men) would use a home-sampling test?
  - 0 Nobody – 100 Everybody
