# Supplementary material for: Intention to use and acceptability of home-based sexual health care among men who have sex with men who previously attended clinic-based sexual health care
Source: Front Reprod Health. 2022 Aug 15;4:967770. doi: 10.3389/frph.2022.967770 (PMC9580753; doi:10.3389/frph.2022.967770)
Supplement: Supplementary file 4 [file Table_4.pdf]

Supplementary Table S4. Questions to determine acceptability of self-sampling STI/HIV testing and online sexual health counseling

- **If you would use a home-sampling test, how would you prefer to get this test? Please select all that apply.**

  Order online and home delivery

  Order over the phone and home delivery

  Get from a friend or sex partner

  Pick up at the STI clinic (GGD)

  Pick up at the general practitioner (GP)

  Pick up at the hospital

  Pick up at an HIV treatment centre

  Get when I am at a sauna, a party or nightlife

  No preference

  Other, please specify…

- **Do you think you are going to need support (help and/or information) when home-sampling testing for HIV or STIs?**

  Yes

  No

  I do not know yet

- **How would you like to get support on home-sampling testing? Please select all that apply.**

  Through written content on a website

  Through a video or footage on a website

  Through an online chat service with a health care professional

Through a webcam consultation with a health care professional

Through a consultation over the phone with a health care professional

Through a friend/sex partner/other acquaintance who have experience with home-sampling testing

Other, please specify…

- **Would you recommend home-sampling tests to other men (who have sex with men)?**

  Yes

  - To a regular partner (relationship)
  - To a regular sex partner (fuckbuddy)
  - To casual sex partner(s) (you know their name)
  - To casual sex partner(s) (you do not know their name)
  - To good friend(s) who are close to me
  - To other friends or acquaintances
  - To chat friends
  - I do not know yet who I would recommend home-sampling tests

  No

<u>Online sexual health counseling</u>

- **"I am … towards remote consultations"**
  0 Negative – 100 Positive

- **If you had the choice, how would you like to get consultations from the STI Clinic of the Public Health Service (GGD)?**

  I would like all consultations to be online/over the phone

  I would like some of the consultations to be online/over the phone, and some on-site: a combination of both

I would not like to have consultations online/over the phone: I want every consultation on-site

- **Which form of remote consultations would you prefer? Please select all that apply.**

  Consultation over the phone

  Online consultation (by chat)

  Webcam consultation (videocall)
