# Supplementary material for: Intention to use and acceptability of home-based sexual health care among men who have sex with men who previously attended clinic-based sexual health care
Source: Front Reprod Health. 2022 Aug 15;4:967770. doi: 10.3389/frph.2022.967770 (PMC9580753; doi:10.3389/frph.2022.967770)
Supplement: Supplementary file 5 [file Table_5.pdf]

Supplementary Table S5. Intention to use a self-sampling STI/HIV test compared between study characteristics with a Kruskal-Wallis H test (N=154)

	Intention self-sampling testing		
	N	Median	*p*
**Ethnicity** [a]			.408
Western	147	86.0	
Non-Western	7	87.0	
**Age** [b]			.726
15 - 42yr	49	90.0	
43 - 54yr	57	82.0	
55 + yr.	48	82.5	
**Education** [a *]			.151
High	97	97.0	
Low	46	80.5	
**No. sex partners** [b c]			.673
0 – 3	58	88.5	
4 – 8	43	81.0	
8 +	53	90.0	
**CAI with casual partner** [c]			.509
Yes	92	81.0	
No	62	89.5	
**HIV status** [*]			.171
Positive	25	99.0	
Negative	126	83.5	
**PrEP use**			.502
Yes	58	80.5	
No	96	89.0	
**Chemsex**			.360
Yes	57	98.0	
No	97	85.0	
**STI/HIV testing** [c]			.297
Yes	93	82.0	
No	61	96.0	

[a] Ethnicity and level of education were based on definitions used by Central Bureau of Statistics (NL) (http://www./cbs.nl). Middle level of education is classified as highly educated.
[b] Age groups and number of sex partners were based on tertile distributions
[c] In the past six months

[*] Education and HIV status do not count to 100 % due to missing educational level of 7.1% and 1.9 % that did not want to declare their HIV status
